# MicroRNA-based regulation of Aurora A kinase in breast cancer

**DOI:** 10.18632/oncotarget.27811

**Published:** 2020-11-17

**Authors:** Adewale Oluwaseun Fadaka, Nicole Remaliah Samantha Sibuyi, Abram Madimabe Madiehe, Mervin Meyer

**Affiliations:** ^1^Department of Science and Innovation/Mintek Nanotechnology Innovation Centre, Biolabels Node, Department of Biotechnology, Faculty of Natural Sciences, University of the Western Cape, Bellville, South Africa; ^2^Nanobiotechnology Research Group, Department of Biotechnology, Faculty of Natural Sciences, University of the Western Cape, Bellville, South Africa

**Keywords:** AURKA, breast cancer therapy, regulatory microRNA, human argonaute, gene expression

## Abstract

The involvement of non-coding RNAs (ncRNAs) in cellular physiology and disease pathogenesis is becoming increasingly relevant in recent years specifically in cancer research. Breast cancer (BC) has become a health concern and accounts for most of the cancer-related incidences and mortalities reported amongst females. In spite of the presence of promising tools for BC therapy, the mortality rate of metastatic BC cases is still high. Therefore, the genomic exploration of the BC subtype and the use of ncRNAs for possible regulation is pivotal. The expression and prognostic values of AURKA gene were assessed by Oncomine, GEPIA, KM-plotter, and bc-GenExMiner v4.4, respectively. Associated proteins and functional enrichment were evaluated by Cytoscape and DAVID databases. Additionally, molecular docking approach was employed to investigate the regulatory role of hsa-miR-32-3p assisted argonaute (AGO) protein of AURKA gene in BC. AURKA gene was highly expressed in patients with BC relative to normal counterpart and significantly correlated with poor survival. The docking result suggested that AURKA could be regulated by hsa-miR-32-3p as confirmed by the reported binding energy and specific interactions. The study gives some insights into role of AURKA and its regulation by microRNAs through AGO protein. It also provides exciting opportunities for cancer therapeutic intervention.

## INTRODUCTION

Breast cancer (BC) is the most frequently diagnosed cancer among females and accounts for most of the cancer-related incidences and mortalities [1] Globally, statistics have shown massive increase in incidence and mortalities from 1.7 million and 521,900 to 8.6 million and 4.2 million in BC, respectively [1, 2]. Approximately one in eight women will be diagnosed with invasive BC in their lifetime and one in 39 women will die from BC [3]. There are increasing attempts in cancer research to correlate molecular subtypes of BC in order to tailor treatment and develop new therapies [4]. Based on BC molecular subtypes, luminal A tumor appears to present better prognosis, have high survival rate and reduced chances of recurrence [5–7]. Currently used markers in the management and prognostic of BC do not equate accurate prediction of how individuals will respond to prescribed treatment of each subtype and their survival thereof. The poor survival and high mortality rates in BC are in part due to lack of effective BC prognostic biomarkers. It is therefore necessary to identify novel biomarkers that can serve as targets for BC therapeutic intervention.

More so, genomic exploration has been employed to identify novel gene regulators or roles of pre-existing gene members in signaling pathways [8]. In the same fashion, this advancement can be deployed in cancer research to sort out genetic alteration in order to tailor therapeutic responses most especially in BC [9].

The human protein kinases are fascinating targets for the discovery of novel cancer therapy due to their crucial role in cancer development and progression, and other related processes such as metabolic diseases [10, 11]. Aurora A kinase (officially: AURKA; aliases: Aurora-2, RKI, and STK15) is a conserved serine/threonine kinase which belong to the superfamily aurora kinase (AK). The AK primarily functions by regulating key cellular functions including mitosis and signaling pathways. The dysfunction of AK may result in mitotic arrest, aneuploidy, and apoptosis. Increased expression or amplification of AURKA is common in most human cancers including BC [12–18]. AURKA has been established as a legit oncogene and thus, a vital therapeutic target in cancer [19]. Its expression has been shown to be modulated by small molecules including microRNAs in cancers [20–23].

Non-coding RNAs (ncRNAs) have been thought to regulate the expression of target genes at post-translational level. The relevance of these ncRNAs particularly microRNAs and ncRNAs as therapeutic targets and delivery strategies in clinical translation were investigated for their involvement in cancer and related diseases. Diverse approaches have both in the past and recently been employed by researches to comprehend the complexity of BC.

Previous studies indicated that miR-32 may be tumor suppressive in nature [24, 25] and its regulatory effect on AURKA have been shown in various cancer subtypes [19, 26–29]. Due to their regulatory role in the expression of tumor associated genes at post-translational level [30, 31], we proposed that human argonaute protein (hAgo2) may regulate the expression of AURKA via hsa-miR-32-3p. Although the complexity of BC subtypes is correlated with molecular and genetic information from tumor cells, prognosis and therapeutic managements are monitored mainly by tumor stage, grade, hormone receptor status and HER2 status. Therefore, *in silico* and molecular docking studies were used to investigate the expression of AURKA gene in relation to its clinicopathological characteristics and the mechanism of regulation through hsa-miR-32-3p assisted hAgo2 in BC.

## RESULTS

### Expression of AURKA in cancers

**Figure 1:**
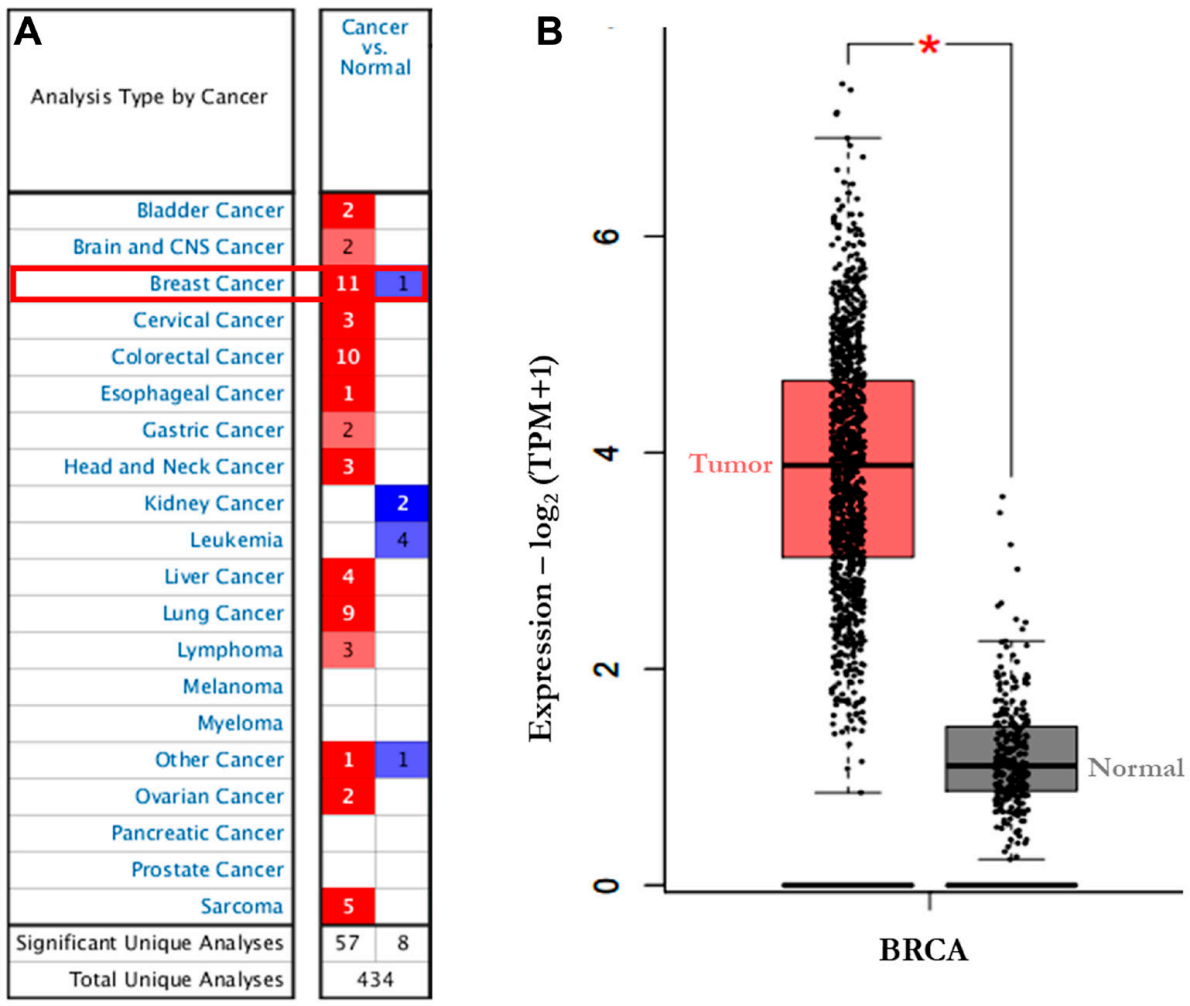
The transcription levels of AURKA in different types of cancers (Oncomine). The graphic demonstrated the numbers of datasets with statistically significant mRNA over-expression (red) or down-expression (blue) of the target gene. (**A**) the expression of AURKA in 20 datasets (Oncomine). (**B**) the expression of AURKA in BC (GEPIA). The threshold was designed with following parameters: Threshold (*p*-value): 1.0 × 10^-6^; threshold (fold change) × 2; and gene rank of top 5%; TPM: Transcript per million. Abbreviations: GEPIA, Gene Expression Profiling Interactive Analysis.

**Figure 2:**
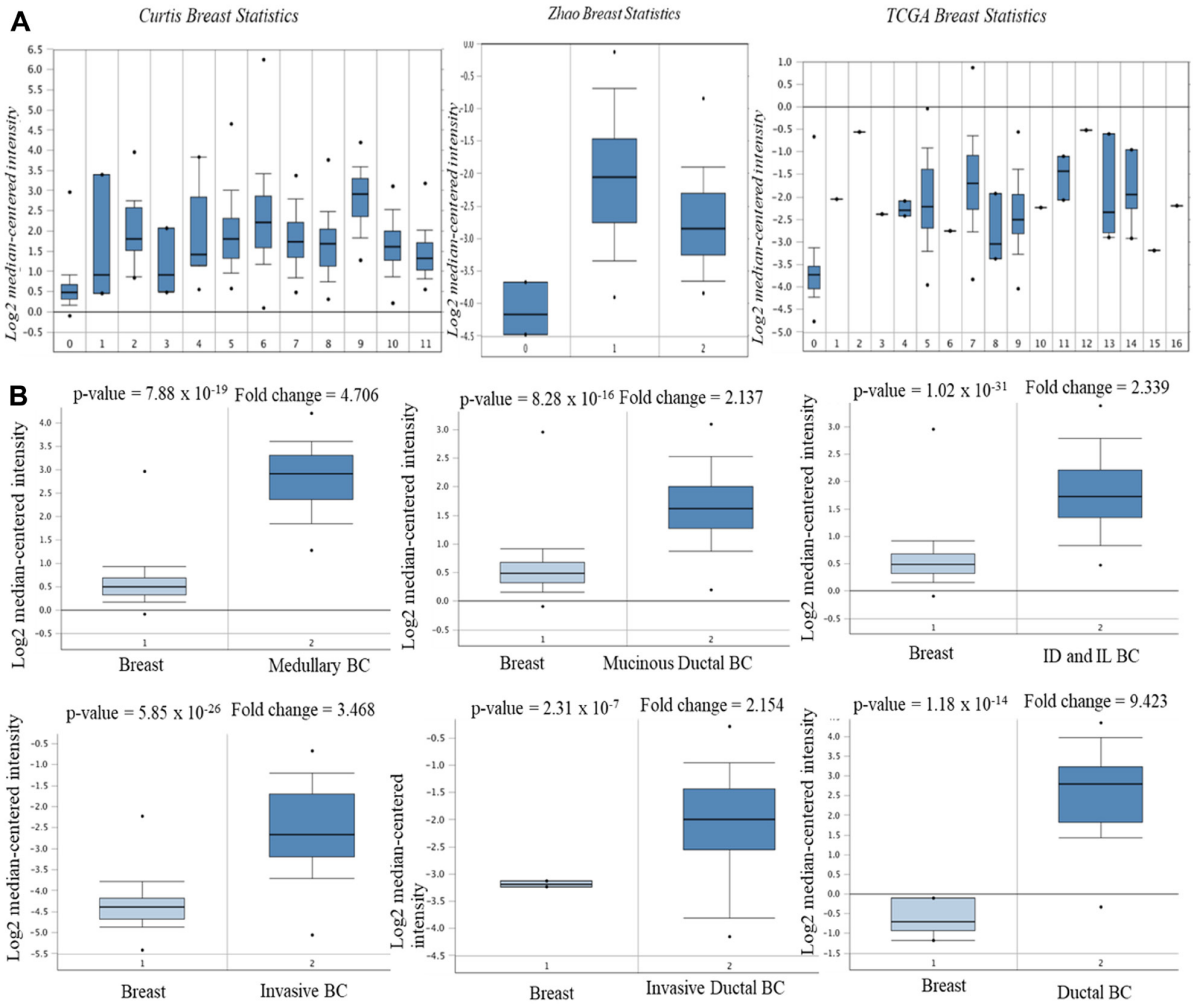
mRNA expression of AURKA in BC subtypes (Oncomine database). The three datasets (**A**) and the BC subtypes (**B**). Note: Curtis Breast Statistics: 0. Breast (B); 1. Benign Breast Neoplasm (BBN); 2. Breast Carcinoma (BC); 3. Breast Phyllodes Tumor (BPT); 4. Ductal Breast Carcinoma *in situ* (DBCi); 5. Invasive Breast Carcinoma (IBC); 6. Invasive Ductal Breast Carcinoma (IDBC); 7. Invasive ductal and invasive lobular breast carcinoma; 8. invasive lobular Breast Carcinoma; 9. Medullary Breast Carcinoma (MBC); 10. Mucinous Breast Carcinoma (MuBC); 11. Tubular Breast Carcinoma (TCB). Zhao Breast Statistics: 0. Breast; 1. Invasive ductal BC; 2. Lobular BC. TCGA Breast Statistics: 0. Breast; 1. Apocrine Breast Carcinoma; 2. Breast Large Cell Neuroendocrine Carcinoma; 3. Ductal Breast Carcinoma; 4. Intraductal Cribriform Breast Carcinoma; 5. Invasive Breast Carcinoma; 6. Invasive Cribriform Breast Carcinoma; 7. Invasive Ductal Breast Carcinoma; 8. Invasive Ductal and Lobular Carcinoma; 9. Invasive Lobular Carcinoma; 10. Invasive Papillary Breast Carcinoma; 11. Male Breast Carcinoma; 12. Metastatic Breast Carcinoma; 13. Mixed Lobular and Ductal Breast Carcinoma; 14. Mucinous Breast Carcinoma; 15. Papilary Breast Carcinoma; 16. Pleomorphic Breast Carcinoma.

Using Oncomine database, AURKA expression was identified in various human cancers, in both hematological malignancies and solid tumors (Figure 1A). The mRNA levels of AURKA were significantly upregulated in BC patients in 11 datasets and down-regulated in 1 dataset. Furthermore, the GEPIA dataset revealed that the mRNA expression level of AURKA was significantly higher in BC tissues than in normal breast tissues (Figure 1B). In Curtis Breast Statistics’ dataset, AURKA was overexpressed compared to normal samples in all breast cancer types (Figure 2). In Zhao Breast Statistics’ dataset, AURKA was overexpressed in invasive ductal breast cancer and lobular BC (Figure 2). In TCGA Statistics’ dataset, AURKA was overexpressed in all the samples (Figure 2) while down-regulation was observed in the Finak Breast Statistics’ dataset in invasive BC with fold change of -11.071 compared to the normal samples. As shown in Table 1, the transcription levels of AURKA in different BC types were also higher than those in normal breast tissues at *P* < 0.05.

**Table 1:** AURKA mRNA expression level in BC compared to normal tissues (Oncomine) in 12 datasets

BC Subtype	Fold-change	*P*-value	Rank (%)	Sample	Source
**Ductal BC**	9.423	1.18 × 10^-14^	1	47	(Richardson *et al.*, 2006)
**Medullary BC**	4.706	7.88 × 10^-19^	1	2136	(Curtis *et al.*, 2012)
**Invasive Ductal BC**	3.168	4.88 × 10^-120^	1	2136	(Curtis *et al.*, 2012)
**Invasive Lobular BC**	2.115	5.80 × 10^-44^	2	2136	(Curtis *et al.*, 2012)
**Invasive Ductal & Invasive Lobular BC**	2.339	1.02 × 10^-31^	2	2136	(Curtis *et al.*, 2012)
**Invasive BC**	2.586	9.96 × 10^-7^	3	2136	(Curtis *et al.*, 2012)
**Mucinous BC**	2.137	8.28 × 10^-16^	3	593	TCGA, 2011
**Invasive Ductal BC**	4.702	5.39 × 10^-53^	1	593	TCGA, 2011
**Invasive BC**	3.468	5.83 × 10^-26^	1	593	TCGA, 2011
**Invasive Lobular BC**	2.351	7.20 × 10^-14^	2	64	TCGA, 2011
**Invasive Ductal BC**	2.154	2.34 × 10^-7^	4	64	(Zhao *et al.*, 2004)
**Invasive Breast Carcinoma Stroma**	–11.071	2.11 × 10_-30_	2	59	(Finak *et al.*, 2008)

Notes: *P*-value was analyzed using the *t*-test. The *P*-values indicate that the difference was statistically significant between the BC and normal tissue group. Abbreviations: BC, breast carcinoma. mRNA levels of AURKA in different subtypes of BC.

### Survival analysis of AURKA in BC

**Figure 3:**
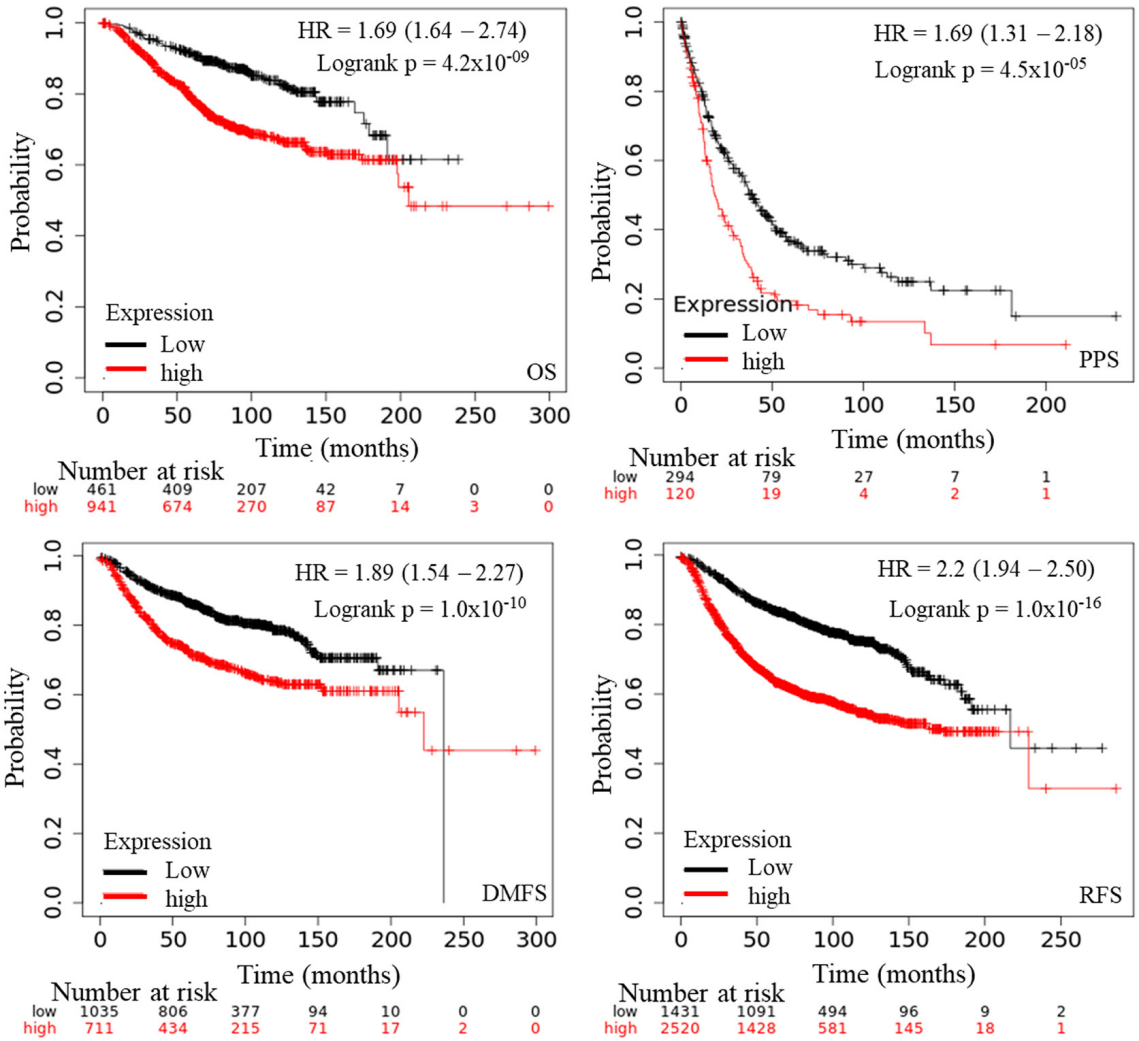
The prognostic value of mRNA level of AURKA in BC patients (Kaplan–Meier plotter). Notes: The OS, PPS, DMFS, and RFS survival curve comparing the patient with high (red) and low (black) AURKA expression in BC were plotted from Kaplan–Meier plotter database as the threshold of *P*-value < 0.05, respectively. Endpoints Affymetrix IDs: 208079_s_at. Abbreviations: OS: overall survival; PPS: progression free survival; RFS: relapses free survival; DMFS: distance metastasis free survival.

**Figure 4:**
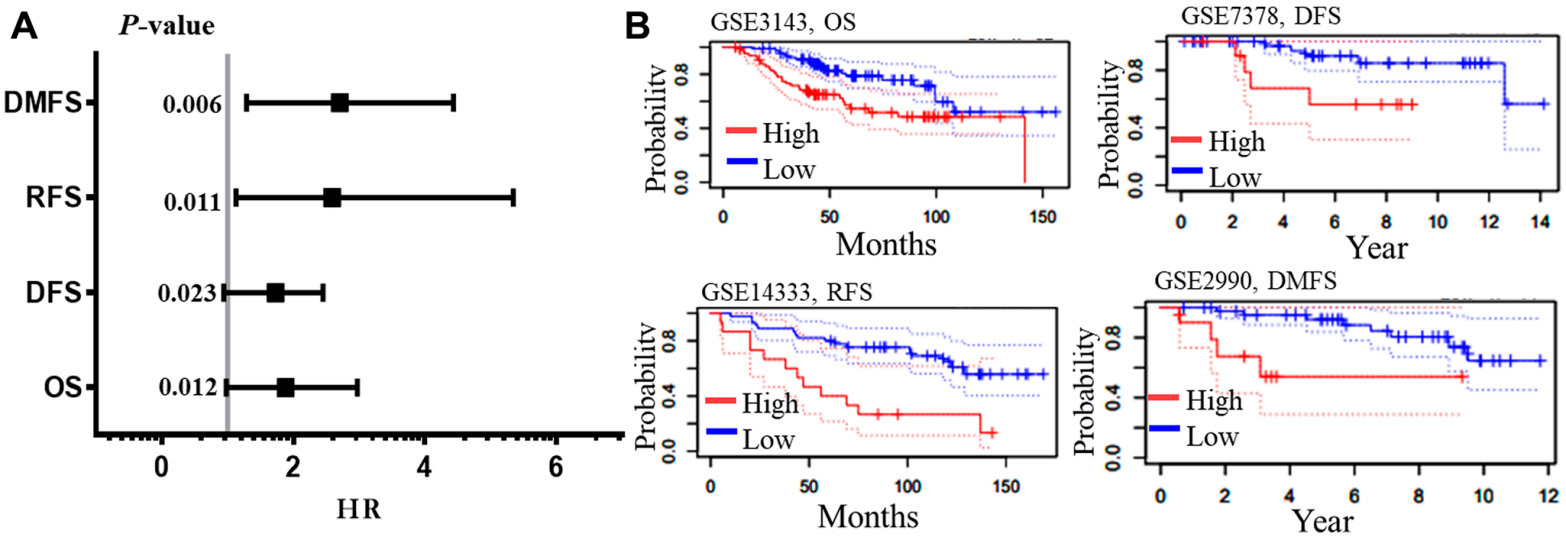
Survival analysis of AURKA in BC. The meta-analysis of the hazard ration with their *p*-values (**A**), and the four end points pictorial images from PrognoScan database. (**B**) The survival curves depict the high (Red) and low (blue) expressions with the datasets and endpoints. Abbreviations: OS: overall survival; DFS: disease free survival; RFS: relapses free survival; DMFS: distance metastasis free survival; HR: hazard ratio.

The prognostic significance of AURKA was assessed in all BC datasets using KM-plotter (Figure 3). The increased AURKA mRNA level was strongly associated with poor overall survival (OS), post-progression survival (PPS), distant metastasis free survival (DMFS) and recurrence-free survival (RFS). To validate this result, PrognoScan database was employed. The PrognoScan revealed that high expression of AURKA was significantly associated with poor OS, DFS, higher risk of distance metastasis and relapse at *p* < 0.05 (Figure 4A and 4B).

### Prognostic values of AURKA in BC patients with different clinicopathological features

The association of AURKA gene with other clinicopathological features (such as; ER, PR, HERS2, lymph node status, TP53 status, as well as the BC’s pathological and clinical grades), and its correlation with these clinicopathological features was assessed in BC (Table 2). The mRNA expression level of AURKA was correlated with OS and DMFS of all patients with BC. In terms of ER status, increased mRNA expression of AURKA was associated with longer DMFS in ER-negative, but high mRNA expression of AURKA was linked to poor OS and DMFS in ER-positive and OS in ER-negative in BC patients. For ER status, increased expression was associated with poor prognosis both positive-ER in OS and DMFS and in negative-ER in OS. Only the ER-positive was related to better survival in DMFS. Conversely, the mRNA levels of AURKA is significantly higher in human epidermal growth factor receptor 2 (HER2) positive BC patients compared with the HER2-negative ones. Although there is significant correlation between the two subtypes, increased expression was associated with good prognosis in HER2-positive in both OS and DMFS while poor survival was observed in HER2-negative in OS and DMFS. For clinical lymph node status in BC patients, high mRNA expression of AURKA was associated with poor OS and DMFS in the subgroups (positive and negative nodal status). The high expression of AURKA mRNA was associated with improved OS and poor DMFS in pathological grade I BC patients; and poor OS and DMFS in pathological grade II BC patients. However, there is no significant association of AURKA with prognosis in grade III BC patients. Mutation of TP53 in cancers, including BC, is very common [32]. Its wild type codes for p53 which is involved in a number of physiological functions such as cellular senescence, metabolism, DNA repair, cell cycle arrest, programmed cell death, and many other processes following cellular stress [33]. Mutated TP53 may cause BC to metastasize as a result of altered p53 protein, which in turn fails to recognize and trigger apoptosis in cells with mutated genes. TP53 mutations are a negative prognostic factor in BC (Varna *et al.*, 2011). Tumors with TP53 mutations are more likely to be aggressive (triple-negative (basal-like) or HER-2-positive (Luminal-B) BC) [34–36]. High mRNA expression level of AURKA was associated with poor OS and DMFS in wide type TP53 BC. However, high expression levels of this gene were not correlated with mutated-TP53-type BC. The welch’s test (bc-GenExMiner) was further used to analyze the subtypes of each clinicopathological features.

**Table 2:** The prognostic values of AURKA in BC patients with different clinicopathological features (Kaplan–Meier plotter: dataset 208079_s_at)

AURKA	Clinicopathological	OS				DMFS			
	features	Case	HR	95% CI	*p*-value	Case	HR	95%CI	*p*-value
	Overall	1402	2.12	1.64–2.74	**4.2 × 10**^–9^	1746	1.87	1.54–2.27	**1.0 × 10**^-10^
ER stat	ER (+)	548	2.2	1.54–3.13	**7.7 × 10**^–6^	664	2.74	1.96–3.82	**8.4 × 10**^-10^
	ER (–)	251	1.33	0.83–2.12	0.23	218	0.54	0.34–0.85	**0.0072**
PR stat	PR (+)	83	4.1	0.1–5.99	**0.02**	192	4.41	1.13–14.83	**0.0088**
	PR (–)	89	2.7	0.96–7.57	**0.05**	154	0.66	0.34–1.27	0.21
HER2	HER2 (+)	129	0.37	0.15–0.9	**0.023**	126	0.42	0.21–0.83	**0.0096**
	HER2 (–)	130	8.33	1.12–62.17	**0.013**	150	3.11	1.31–7.38	**0.0068**
LN stat	LN (+)	313	1.59	1.05–2.42	**0.029**	382	1.78	1.21–2.63	**0.0031**
	LN (–)	594	2.23	1.53–3.25	1.7 × 10^–5^	988	2.3	1.74–3.02	**1.0 × 10**^-9^
Grade	I	161	4.888	1.41–16.9	**0.0057**	188	4.5	1.66–12.26	**0.0013**
	II	387	2.02	1.31–3.11	**0.0012**	546	2.2	1.56–3.12	**4.8 × 10**^-6^
	III	503	1.35	0.97–1.87	0.074	458	1.22	0.86–1.73	0.27
TP53	Mutated	111	0.42	0.12–1.39	0.14	83	0.37	0.11–1.25	0.095
	Wide type	187	3.36	1.31–8.63	**0.0075**	109	2.8	1.28–6.12	**0.0071**

Notes: *P*-value was analyzed using the survival analysis test. The bold font indicates that the difference was statistically significant. Abbreviations: ER stat: Estrogen status; PR stat: progesterone status; LN stat: lymph node status; +: positive; –: negative; TP53 mut: HER2: human epidermal growth factor receptor 2; TP53 mutation; OS: overall survival; DMFS: distance metastasis free survival; BC: breast cancer. Clinicopathological association of AURKA with prognosis in BC.

### Genetic alterations of AURKA and clinicopathological parameters in BC patients

**Figure 5:**
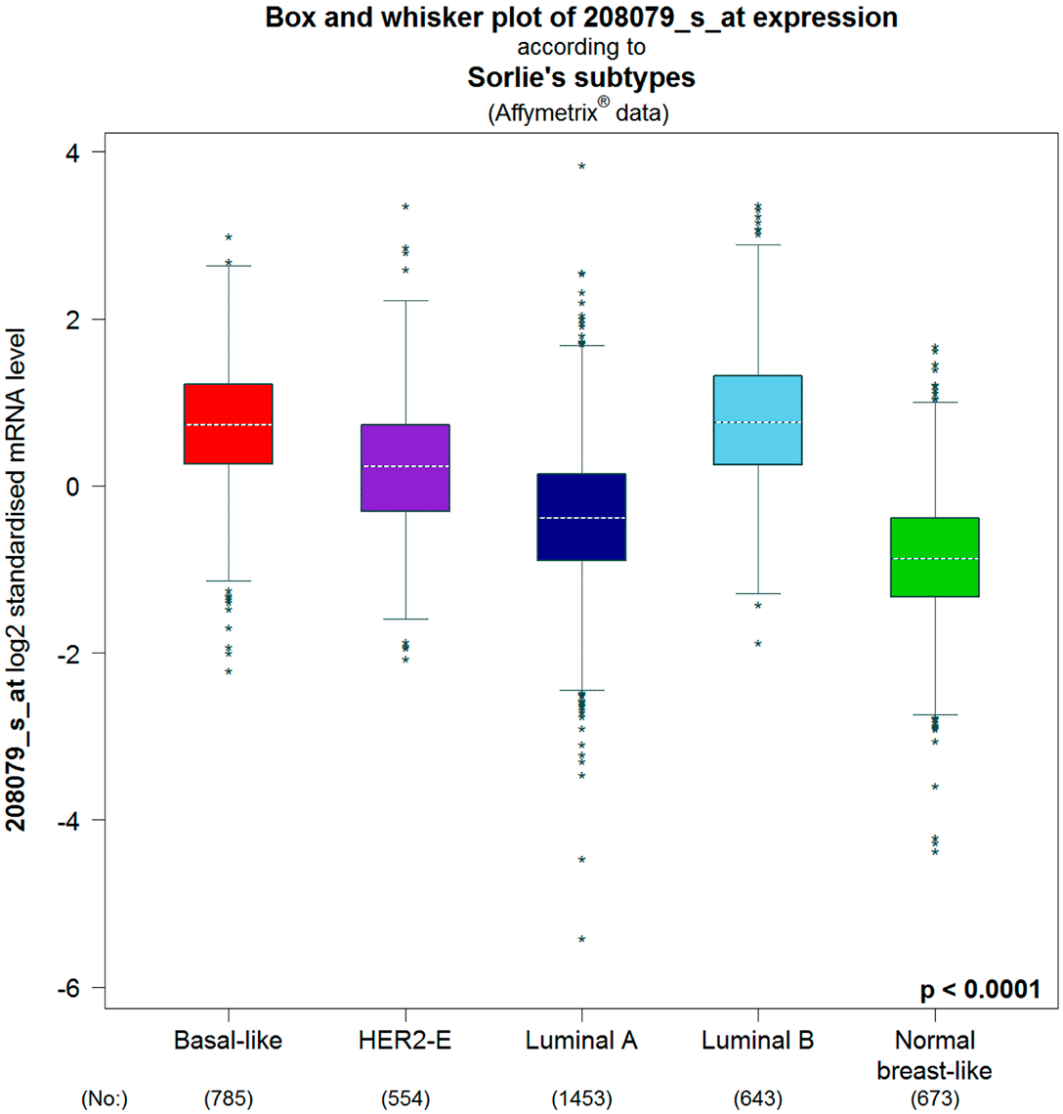
Intrinsic molecular subtype of AURKA in BC patients (bc-GenExMiner v4.4). The box plots are based on Intrinsic molecular subtype of AURKA. Correlation between AURKA expression and genetic information in BC patients. The significant different between groups was assessed by Welch’s test to generate *p* value, along with Dunnett-Tukey-Kramer’s and the *P*-value was set at 0.05.

As depicted in Table 3, the transcription levels of AURKA were compared by bc-GenExMiner using the welch’s test among BC patients associated with some clinicopathological parameters [37]. These parameters include the age of the patient, together with statuses on the disease nodal, absence or presence of BC receptors, TP53, and so on. For age criterion, high expression of AURKA was significantly associated with patients above 51 years compared to patients below the age of 51 years. Also, BC patients with positive nodal status, mutated-TP53, basal-like and triple negative BC (TNBC) status had higher AURKA mRNA compared to those with negative-nodal, wild type-TP53, not-basal-like and not-TNBC statuses (Table 3). Receptor statuses (PR and ER) were negatively correlated with the AURKA expression. On the contrary, the AURKA mRNA levels were significantly increased in BC patients with higher expression levels of HER2 compared to the HER2-negative subtype (Table 3). In Sorlie’s intrinsic molecular subtypes, the components were associated with the higher mRNA level of AURKA but the basal-like and luminal B has no difference with respect to elevated level of AURKA (Figure 5).

**Table 3:** The relationship between mRNA expression of AURKA and clinicopathological features of BC

Variables	AURKA		
	Case	mRNA	*p*-value
**Age**			0.0001
≥ 51	1387	↑	
≤ 51	1974	—	
**Nodal status**			0.0221
+	1574	↑	
–	2215	—	
**ER**			0.0001
+	3255	↓	
–	1177	—	
**PR**			0.0001
+	1335	↓	
–	917	—	
**HER2**			0.0001
+	286	↑	
–	1711	—	
**TP53 Status**			0.0001
Mutation	247	↑	
Wild Type	523	—	
**Basal-Like Status**			0.0001
Basal-Like	962	↑	
Not Basal-Like	3549	—	
**Triple-Negative Status**			0.0001
TNBC	416	↑	
Not	3439	—	

Note: ER: estrogen; PR: progesterone; HER2: human epidermal growth factor receptor 2; (+): positive; (–): negative; TP53: tumor protein 53; TNBC: triple-negative BC. The significant different between groups was assessed by Welch’s test to generate *p* value, along with Dunnett-Tukey-Kramer’s and the *P*-value was set at 0.05.

### Gene association of AURKA

**Figure 6:**
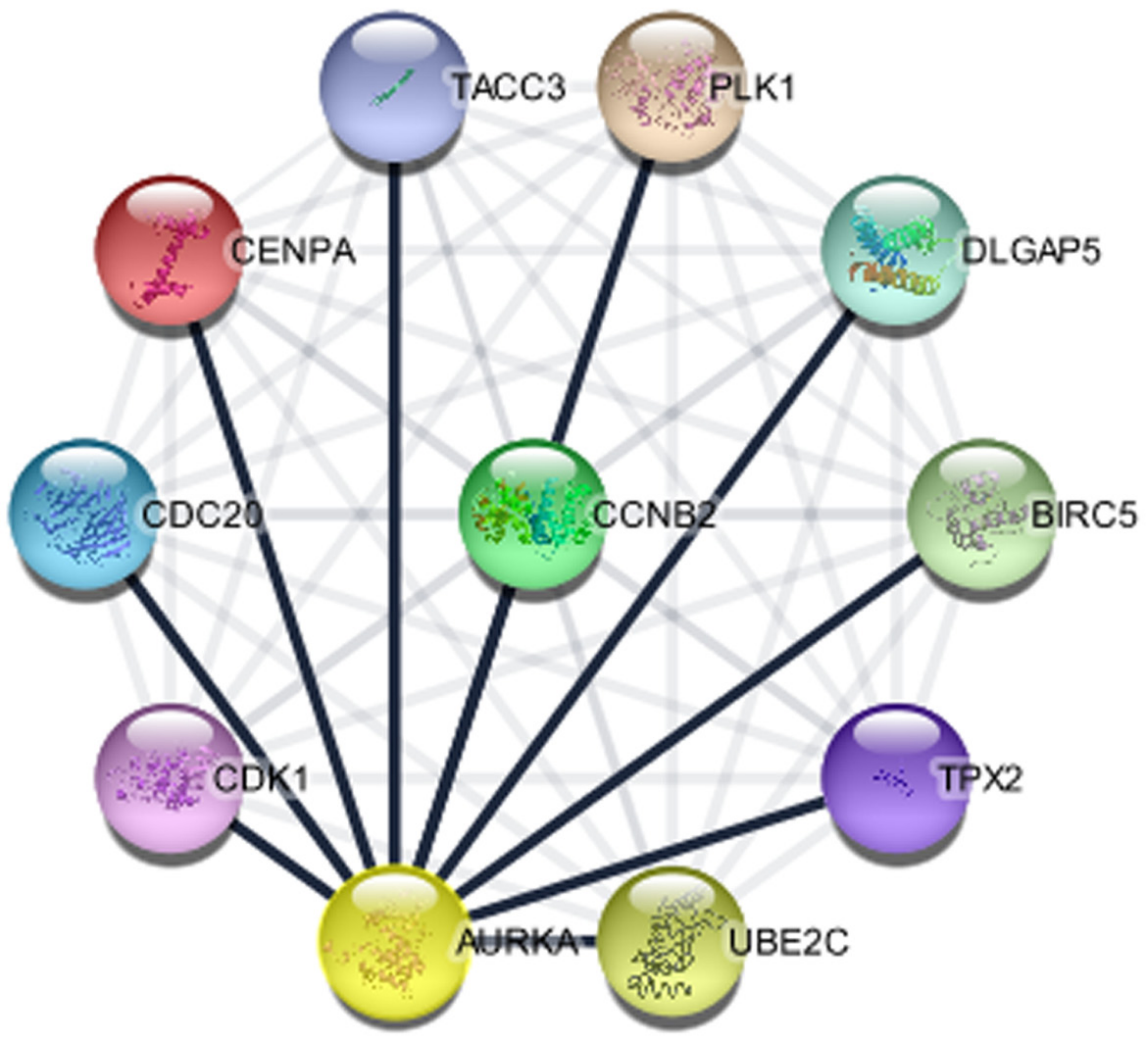
The protein interaction network of genes associated with AURKA (STRING in Cytoscape).

The importance of PPis have been reported in biological processes and cellular function in both prokaryotes and eukaryotes including humans (normal and disease states) [38]. Identification of protein partners for AURKA can be an important lead to unravel the mechanisms of action for diseases typically cancer and further generate therapeutic drugs and treatment advancement. Numerous experimental approaches have been used for the discovery of interacting genes but the processes are demanding with limited outputs. However, computational approach has been increasingly used to validate and predict hidden protein partners. This study identified 10 proteins associated with AURKA genes through curated database entries, experimental validation, text mining, co-expression and or protein homology since proteins are constantly regulated and rarely function in isolation (Figure 6). These genes are crucial to the roles and specific molecular network of AURKA in disease, most importantly BC. The 10 associated proteins identified for AURKA at *p* < 0.05 include: Polo like kinase 1 (PLK1), centromere protein A (CENPA), DLG associated protein 5 (DLGAP5), TPX2, microtubule nucleation factor (TPX2), aurora kinase A (AURKA), baculoviral IAP repeat containing 5 (BIRC5), cell division cycle 20 (CDC20), cyclin B2 (CCNB2), cyclin dependent kinase 1 (CDK1), transforming acidic coiled-coil containing protein 3 (TACC3), ubiquitin conjugating enzyme E2 C (UBE2C), have a PPI enrichment *p*-value of 2.74 × 10^-13^, average node degree of 10 and average local clustering coefficient of 1.0. Further studies to give insights into the relationship of each of the identified genes with respect to AURKA expression in BC, is highly recommended.

### Functional enrichment and pathway analysis

The discovery of pathways and specific processes that are significant with factors regulating activities to genes of interest is important in cancer research. To identify functional categories and characteristic biological attributes of associated genes, gene oncology (GO) enrichment analysis was performed using DAVID database. DAVID gives a high-throughput and attractive data collection condition, and merge the functional genomic annotations with intuitive graphical representations encouraging the transition between genomic information and the biological meaning. The GENETIC_ASSOIATION_DB_DISEASE analysis was employed to evaluate AURKA gene targets associated with BC from the setup installation file of the genes from STRING database. Additionally, GO provides classifications of genes in relation to their molecular and cellular structures and functions [39].

The three categories of GO terms include the biological process (BP), cellular component (CC), and molecular function (MF). The KEGG pathway in DAVID also contains adequate information of known metabolic and regulatory pathways and accelerates the mapping of genes to KEGG pathways for systemic analysis of gene functions [40].

To explore the functions as well as the pathways crucial to the associated genes in relation to BC, GO and KEGG pathway enrichment analyses were carried out for the protein partners as presented in Table 4. The *p*-value is the probability that the chosen gene for any of the three GO term categories occurred by chance. Therefore, a low *p*-value corresponds to a greater likelihood of significance set at the false discovery rate (FDR) <0.01. The AURKA associated proteins are mainly involved in all three GO term categories in BP ontology (cell division), MF ontology (protein binding), and CC ontology (spindle). KEGG pathway indicated that the AURKA and associated genes were mainly enriched in pathways such as cell cycle, progesterone-mediated oocyte maturation, oocyte meiosis, and p53 signaling pathway.

**Table 4:** Functional and pathway enrichment analysis of AURKA and its associated genes (DAVID)

Category	Term	Count	%	*p*-value
**GOTERM_BP_DIRECT**	cell division	8	72.7	1.80 × 10^-10^
**GOTERM_BP_DIRECT**	mitotic nuclear division	7	63.6	2.00 × 10^-09^
**GOTERM_BP_DIRECT**	G2/M transition of mitotic cell cycle	6	54.5	8.20 × 10^-09^
**GOTERM_BP_DIRECT**	anaphase-promoting complex-dependent catabolic process	5	45.5	9.30 × 10^-08^
**GOTERM_BP_DIRECT**	regulation of ubiquitin-protein ligase activity involved in mitotic cell cycle	4	36.4	2.70 × 10^-07^
**GOTERM_CC_DIRECT**	spindle	5	45.5	3.80 × 10^-07^
**GOTERM_CC_DIRECT**	microtubule cytoskeleton	5	45.5	6.20 × 10^-07^
**GOTERM_CC_DIRECT**	spindle microtubule	4	36.4	1.60 × 10^-06^
**GOTERM_CC_DIRECT**	nucleoplasm	9	81.8	9.90 × 10^-06^
**GOTERM_CC_DIRECT**	spindle pole	4	36.4	2.40 × 10^-05^
**GOTERM_MF_DIRECT**	protein binding	11	100	1.50 × 10^-03^
**GOTERM_MF_DIRECT**	anaphase-promoting complex binding	2	18.2	3.50 × 10^-03^
**GOTERM_MF_DIRECT**	ATP binding	5	45.5	8.30 × 10^-03^
**GOTERM_MF_DIRECT**	protein kinase activity	3	27.3	1.80 × 10^-02^
**GOTERM_MF_DIRECT**	protein serine/threonine kinase activity	3	27.3	2.00 × 10^-02^
**KEGG_PATHWAY**	Oocyte meiosis	5	45.5	2.20 × 10^-06^
**KEGG_PATHWAY**	Cell cycle	4	36.4	1.90 × 10^-04^
**KEGG_PATHWAY**	Progesterone-mediated oocyte maturation	3	27.3	3.20 × 10^-03^
**KEGG_PATHWAY**	p53 signaling pathway	2	18.2	6.60 × 10^-02^

Note: BP: biological process; CC: cellular component; MF: molecular process. Gene enrichment.

### Receptor and ligand structure preparation and docking

**Figure 7:**
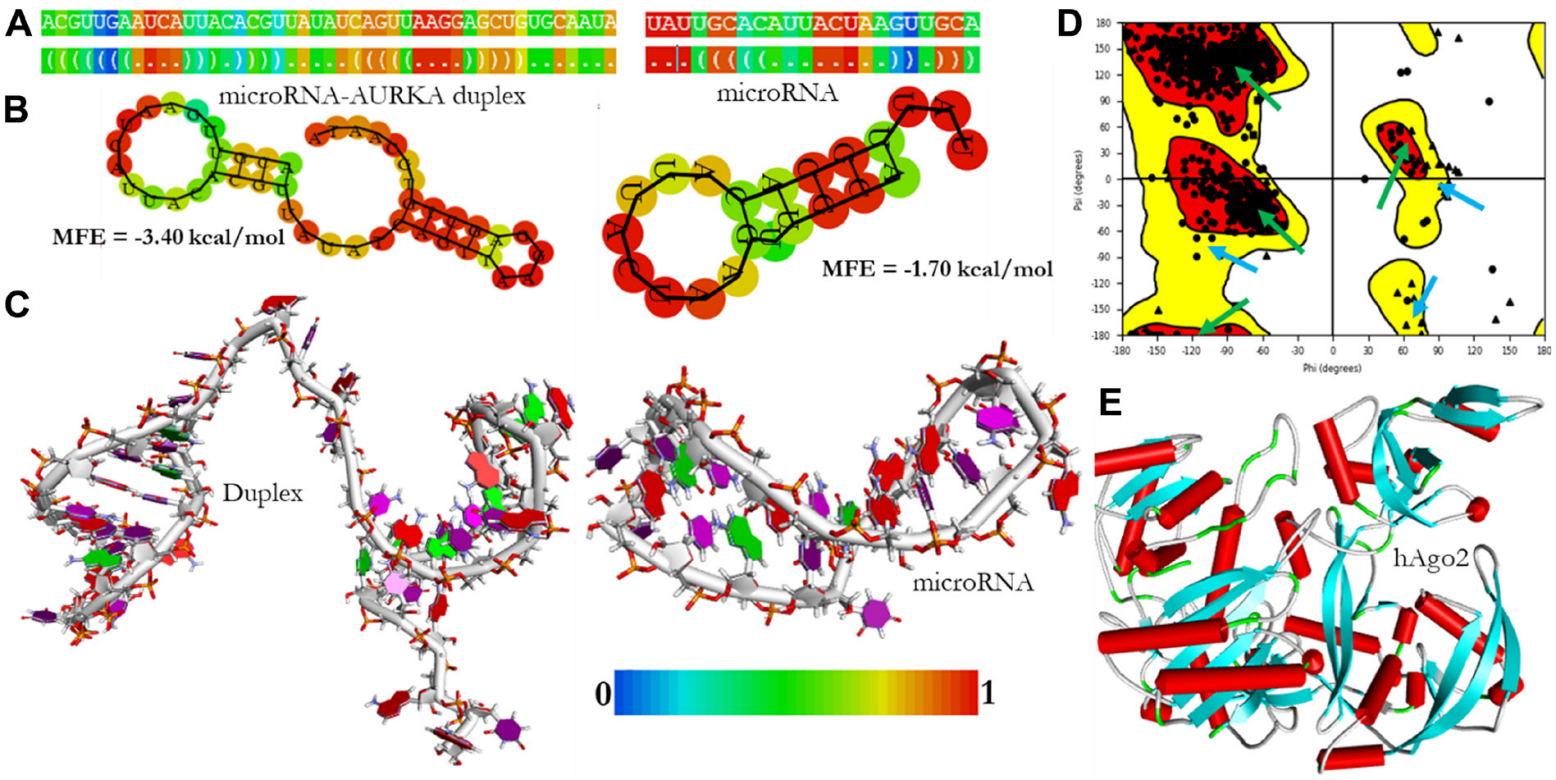
The structural models of microRNA and microRNA-AURKA duplex. The dot-bracket notations are colored by base-pairing probability (**A**), the secondary structures are colored by positional entropy (**B**), the 3D structures were modeled and visualized by DSV v19 (**C**), the Ramachandra plot of the prepared hAgo2 receptor was verified by PDBSum PROCHEK. The area marked with green arrows are residues in the most favored region while the regions marked with teal arrows are residues in additional allowed region (**D**) and the 3D model of the prepared hAgo2 was done by Maestro v12.2 (**E**).

The hAgo2, microRNA, and microRNA-AURKA were considered for the molecular docking analysis in order to evaluate the mechanism of regulation of AURKA in BC (Figure 7). The concept of ligand preparation was to produce corresponding low energy 3D structures from the format generated from RNA-composer with the option to expand each input structures by generating variation on the ionization states, tautomers, stereochemistry and ring conformation (Figure 7A and 7B). The ligands (hsa-miR-32-3p and microRNA-AURKA duplex) were prepared using LigPrep, a module in Maestro (Figure 7C). The receptor, hAgo2 was prepared and verified by Schrodinger suite and PDBSum PROCHEK, respectively (Figure 7D and 7E). Briefly, Protein preparation module in Schrodinger (Maestro v12.2) was employed to ascertain the readiness of the protein for docking by the following steps: (a) Pre-processing (basic task): Assignment of bond orders, filling of missing side chains and loops by prime, and deletion of water molecules beyond 5 Å from het groups; (b) Review and modification: inspection and correction of ionization and the tautomeric state of the het groups present in the receptor using Epik at a target pH range of 7.0+/–2.0; (c) Refinement: Optimization of the orientation of hydrogen bond network (PROPKA at pH 7.0) and structural restrained minimization by force field Optimized Potentials for Liquid Simulations (OPLS_2005) at root mean square deviation (RMSD) of 0.30 Å. As shown in Figure 7D, the crystal structure of hAgo-2 which was used in this study was stable and obeyed theoretical predication.

### Molecular docking analysis

#### Docking analysis

**Figure 8:**
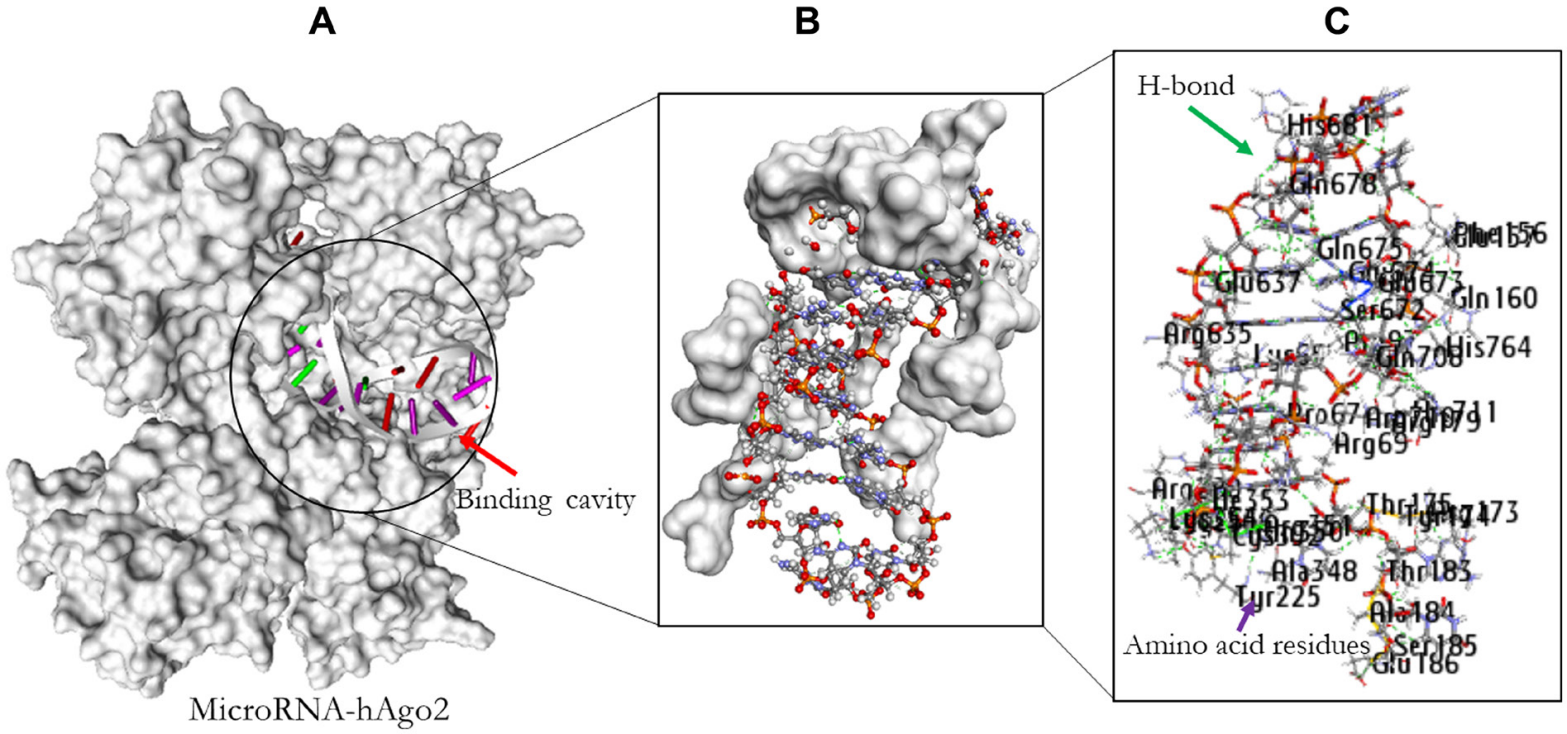
Molecular docking result of miR-32 and hAgo2. The binding position of the miR-32 in the pocket of hAgo2 (**A**); Residual amino acids participating in their interaction (**B**); Amino acid residues (**C**).

**Figure 9:**
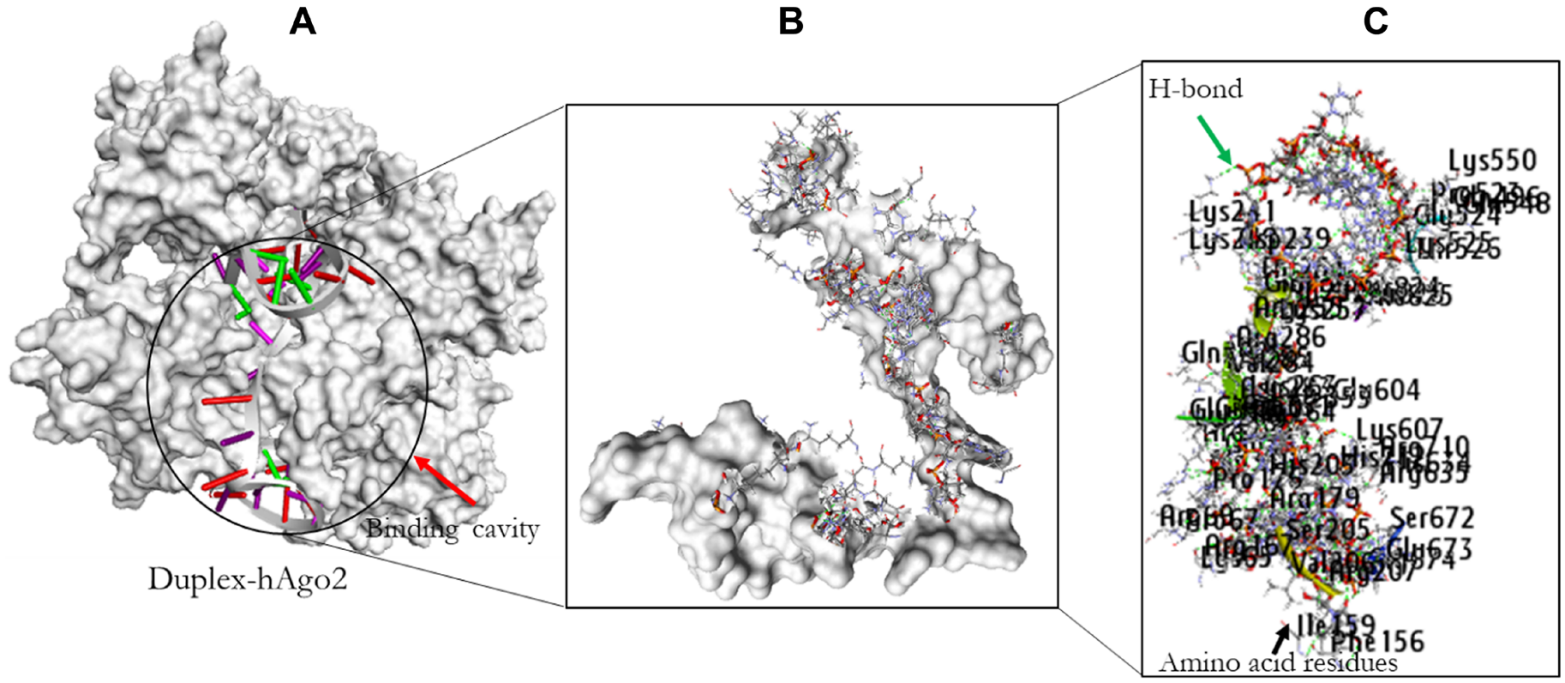
The docking result of miR-32-AURKA and hAgo2. The binding position of the complex in the pocket of hAgo2 (**A**); Residual amino acids participating in their interaction (**B**); Amino acid residues (**C**).

PatchDock web-based docking algorithm was employed for the docking profile of AURKA and hAgo-2 alongside miR-32-3p. The PDB file of hAgo-2 protein and the ligands were used as inputs with clustering RMSD of 1.5 Å. The generated complexes were based on their geometric shape complementarity score. The results with the highest score were chosen for each of the ligands and hAgo-2 (Table 5). The binding affinity of the complex was assessed through their binding scores, and interacting amino acid residues between the ligands and the receptor protein (Tables 6 and 7 and Figures 8 and 9). The result revealed that hAgo-2 protein formed 10 pairs of hydrogen bonds and 3 pairs of Pi interactions with miR-32-3p-AURKA duplex with 59 amino acids involved in the binding cavity of the hAgo-2 protein compared to the 40 amino acids residues found in the pocked of hAgo-2 protein when interacting with miR-32-3p and six pains of hydrogen bonds which demonstrated the complexes are highly stable.

**Table 5:** The docking scores between microRNA, microRNA-AURKA duplex and hAGO-2 protein

Category	Score	Area	ACE	No of AAs
**miR-32-hAgo2**	16038	3113.20	–434.34	40
**Duplex-hAgo2**	24104	4130.40	–379.12	59

Note: ACE: Atomic contact energy; Duplex-hAgo2: microR-32-AURKA-hAgo2; hAgo2: human argonaute 2. The score indicates the geometric shape complementary score and atomic contact energy (ACE) score generated for microRNA-hAgo2 complex and duplex-hAgo-2 complex.

**Table 6:** Molecular docking analysis results of the ligands and receptor with participating aa residues (3.5 Å)

Category	AA involved	Aromatic AA	Hydrophobic AA	AA-H-bonds
**microRNA-hAgo2**	40a	PHE156	LEU265	SER828
		TYR174	ILE353	GLU821
		THR357	VAL237	THR830
		TYR225	LEU238	ASP823
			VAL256	LYS550
			ILE353	ASP499
			LEU356	HIS829
			ALA603	
			ALA825	
**Duplex-hAgo2**	59a	PHE156	ILE159	ARG69
		THR526	ILE353	ARG287
		THR285	VAL206	GLU826
		TYR338	VAL284	ARG286
			ALA825	ASP252
				LYS65
				SER828
				SER824
				GLU821
				GLU821
				PRO523

AA; amino acid. Total hydrophobic residual amino acid involved in docking interaction.

**Table 7:** Hydrogen bonds and their respective residues between the ligands and receptor within the distance of 2 Å

Complex	AA residue	Atoms	Nucleic acid residue	Distance
MicroRNA-hAgo2	SER828	HG - O2	C21	1.9
	GLU821	O - H3	U4	1.9
	THR830	H - OP2	A22	1.3
	ASP823	O - H22	G5	1.9
	LYS550	HE2 - O5′	A15	1.9
	ASP499	OD1 - H5′′	U4	2.0
	HIS829	O - H8	A22	1.6
Duplex-hAgo2	ARG69	H – O4	U39	1.7
	ARG287	HE - O2	U22	2.0
	GLU826	O - HO2′	G18	1.9
	ARG286	HD2 - O3′	U22	1.4
	ASP252	OD2 - H1′	U19	1.7
	LYS65	O – H5	U37	2.0
	SER828	HB2 - O2′	G18	1.8
	SER824	OG - H1′	U4	2.0
	GLU821	OE1 - H2′	U4	1.9
	GLU821	OE2 - H5′	U5	1.6
	PRO523	O - H8	A7	1.9

Note: AA: amino acid. Criteria, such as strong hydrophobic amino acids together with aromatic amino acids, are important to binding interactions in terms of the stability between protein receptors and their ligands.

### DISCUSSION

The process of carcinogenesis and therapeutic responses present a terrific challenge to favorable therapeutic outcome [41]. However, identifying cancer specific targets can pave the way for effective treatment options against specific cancer types [42]. Computational approaches are extensively used to investigate molecular and genetic mechanism of cancer progression by identifying lead genes and the abnormal regulatory pathways of disease [43]. In cancer research, these methods have revealed molecular targets for therapeutic intervention and cancer mechanisms (development, progression, or metastasis) in a systematical, accurate and effective manner [44]. Additionally, due to cancer complexity, it is crucial to investigate the molecular basis and identify significant therapeutic targets of this disease. Recently, researches aimed at identifying the mode of action of BC have increased. Numerous therapeutic interventions have been developed [45]. Nevertheless, these strategies induce a range of therapeutic responses and resistance can develop in BC patients, therefore novel therapeutic intervention is desirable.

Due to the importance of microRNAs in carcinogenesis, they suggest potential alternative therapeutic targets for cancer [46]. Specifically, Polini *et al.* [47] revealed the tumor suppressive role of miR-193a and further suggested that their expression could be attributed to novel treatment for cutaneous melanoma patients.

In this study, the role of AURKA gene expression with clinicopathological details was investigated using *in silico* and molecular docking study and proposed that the expression of AURKA gene may be regulated by miR-32-3p through RNA induced silencing complex in BC. AURKA is a serine/threonine kinase involved in the regulation of mitotic chromosomal segregation and centrosome function. The conservation of AURKA gene has been previously reported in various species including human [48]. Based on its critical role in cancer biology, it may be of great importance to evaluate the status for selecting the most effective therapeutic options most especially in BC.

To evaluate whether a gene have therapeutic target potential, it often needs to be uniquely expressed or highly expressed in tumors than in most normal tissues. Gene expression profiles of 434 total unique analyses in Oncomine were employed to perform bioinformatics analysis in this study. The expression of AURKA was significantly over-expressed in 11 out of 12 BC datasets. The overexpression could be correlated with high occurrence of BC [49, 50]. This result was validated by GEPIA and the mRNA expression level of AURKA was found to be significantly upregulated in BC than normal breast tissues. Studies have shown that AURKA is involved in multiple mechanisms-associated with cancer initiation [51]. AURKA overexpression was also suggested to increase the expression of MMP-2, MMP-7, MMP-9, leading to tumor metastasis by degrading extracellular matrix proteins [52–54]. Zhong *et al.* [55] identified AURKA as potential therapeutic targets in glioblastoma-bearing rats. Therefore, AURKA is an attractive target for cancer therapy.

The transcription levels of AURKA in different BC types were remarkably higher than those in normal tissues, and associated with poorer OS, PPS, DMFS, RFS, and DFS. As evident, the overall clinicopathological features in this study shows that overexpression of this gene was correlated with poor survival. Interestingly, high levels of AURKA was associated with poor OS and DMFS in ER status, PR status, HER2 status, grade stage I and II, and in wide-TP53-type BC patients.

Protein-protein interaction (PPi) network analysis demonstrated other 10 significant genes correlated with AURKA. To further clarify the mechanism of AURKA, a network was constructed for AURKA and the 10 neighboring genes. GO and KEGG analyses indicated that these genes were mainly enriched in a number of GO terms. The functional and pathway enrichment analyses were highly consistent with the findings that p53 signaling, cell cycle and cell division ontology functions were regulated abnormally in BC [56]. This study contributed to the growing evidence regarding the correlated signaling pathway of AURKA which could offer great opportunity into the development of biomarkers for the diagnosis and prognosis of BC.

Phosphorylation/dephosphorylation have been reported as the two predominant mechanisms regulating AURKA [57]. The gene was phosphorylated at three amino acid residues, Serine-53, Threonine-295, and Serine-349. Mutation in the amino acid residues Threonine-295 and Serine-349 is associated with reduced activity. Phosphorylation of Threonine-295 is required for protein kinase (PKA) activation which then phosphorylate (Threonine-295) and activate the kinase *in vitro* [58]. Protein phosphatase-1 negatively regulates Aurora A by dephosphorylating T288 [59]. Additionally, studies show that ncRNAs regulate gene expression by guiding Argonaute proteins to complementary sites on target RNA molecules [60–63]. The expression of microRNAs has been reported in cancers [64–69]. Specifically, microRNAs such are miR-346 and miR-361-3p were suggested to modulate the expression of PSA, TMPRSS2 and DRG1 in prostate cancer [70].

The first microRNA to be reported in the dysregulation of BC was in 2005 [71]. The miR-155 expression was upregulated in BC, its expression was associated with clinicopathological markers, tumor subtype, and poor survival rates [72, 73]. MiR-665 expression was also reported to predicts poor survival and promotion of tumor metastasis by targeting NR4A3 in BC [74]. In addition, deletion of CRISPR/Cas9 suggested the oncogenic roles of miR-23b and miR-27b in BC [75]. Mature microRNAs assist AGO by guiding the complex to target sites in mRNAs that are partially complementary to the microRNA sequence (the seed region) [76], and induce repression of gene expression at the level of mRNA stability or translation [77]. Several proteins interfere with mRNA degradation and translational repression, some of them are necessary components of the RNA induced silencing complex (RISC) that transports those microRNAs to complementary sites within mRNA [78]. The human AGO-2 is a member of the AGO subfamily that is commonly expressed and associate with microRNAs. Although different classes of ncRNAs have different biogenesis pathways and exert diverse functions, all of them must associate with any AGO subfamily for activity [77].

Expression of AURKA among other genes was directly inhibited by miR-186 in neuroblastoma cells [79]. The epigenetic regulation of AURKA by miR-4715-3p has also been reported in gastrointestinal cancers using *in silico* prediction [23]. MicroRNA-490-3p suppresses hepatocellular carcinoma cell proliferation and migration by targeting AURKA [80]. Importantly, the expression of miR-32-3p has been reported to suppress AURKA but the mechanism of action deserves closer attention at both atomic and molecular levels [19]. The possible regulation of AURKA expression was investigated at both atomic and molecular level by evaluating the binding interaction between miR-32-hAgo-2 complex and miR-32-AURKA duplex bound hAgo-2 using molecular docking study. The pool of microRNAs targeting AURKA was assessed by TargetScan. Furthermore, their minimum folding energy as well as their scores were carried out by miRTarBase database. The result showed that miR-32 has the highest score of 164.00 and the lowest minimum energy of -14.20 kcal/mol. This microRNA was further evaluated for its binding activity against AURKA gene using molecular docking analysis. For the secondary folding analysis, determined by the RNAfold, the minimum folding energy (MFE) of the microRNA and microRNA-AURKA duplex were -1.70 kcal/mol and -3.40 kcal/mol, respectively. Three properties (composition, sequence length, and conformation) have been reported to be significant to RNA molecule [81]. Trotta [81] reported that longer sequences are more stable due to their ability to form stacking and hydrogen bond interactions. Sequence arrangement is also another determining factor that affects the folding structure stability. The low MFE of the microRNA was justified by the sequence length of 22 nucleotides and the number of loops formed. The duplex sequence length of 45 nucleotides and extension of loops, are great advantages to confer stability for possible regulation.

In nature, the stability of molecular binding interactions between ligand and its corresponding receptor depends largely on specific amino acids involved (hydrophobic and aromatic amino acid). The interaction between miR-32 and miR-32-AURKA duplex and the human argonaute protein were evaluated through these aforementioned amino acid residues observed in the binding cavity within specific distance. The result revealed the presence of specific interactive residues of hAgo-2 involved in binding of the ligands. Also hydrogen bonds were observed between the interaction atoms of the receptor and the ligands. These interactions taken together, may be important and thus confer regulation of AURKA gene most especially in BC. Furthermore, the number of hydrogen bonds involved in the interacting atoms of hAgo-2 and miR-32-AURKA complex together with the amino acid residues involved in binding, and number of important amino acids such as hydrophobic and aromatic amino in the cavity of hAgo-2 are significantly higher compared to the binding interaction observed in miR-32-hAgo-2 complex. This could therefore, confer stability to the binding of hAgo-2 to AURKA for better regulation.

### MATERIALS AND METHODS

### Expression analysis of AURKA

Web-based data-mining platforms were used to evaluate the expression (Oncomine) of AURKA and further validated using Gene Expression Profiling Interactive Analysis (GEPIA-2) database. Oncomine is a database that contains cancer microarrays from genome-wide expression analyses [82], available at https://www.oncomine.org/. Expression of AURKA was assessed in twenty cancer types and their normal counterparts, and also compared among BC subtypes relative to normal clinical mRNA datasets. Only expression that showed a 2-fold difference between cancer and normal tissues, a *p*-value of 1.0 × 10^-6^, and a priority of 5% were considered.

The mRNA levels of AURKA in BC at a *p*-value less than 0.05 were validated by GEPIA-2 database at http://gepia2.cancer-pku.cn/#analysis. TCGA and GTEx datasets were used to match the tumor as normal data at the cutoff of |Log2FC|of 1.

### Prognosis and expression correlation of AURKA

The statistical exploratory database (Breast cancer Gene expression Miner v4.5) assessed at http://bcgenex.centregauducheau.fr/BC-GEM/GEM-requete.php consists of BC transcriptomic data (10, 0001 DNA microarrays and 4, 712 RNA-seq) [37, 83]. This software offers the possibility to explore expression of genes of interest in BC and the statistical analyses are categorized as correlation, expression and prognosis. This tool was used to study the AURKA expression in BC, the correlation between AURKA mRNA levels and clinicopathological features in BC clinical dataset (Probe set ID: 208079_s_at).

### Survival analysis of AURKA in BC

The Kaplan-Meier database (KM-plot) is a manually curated web-based tool handled by a PostgreSQL server assessed at https://kmplot.com/analysis/index.php?p=service&cancer=breast. This plotter is composed of gene expression and clinical data, and is used to evaluate the prognostic value of a biomarker. The expression of AURKA gene in BC patients in relation to survival rate was analyzed by KM-plot as a function of log-rank *p*-value, and the hazard ratio of 95% confidence intervals. The database can assess 54, 000 cancer-linked genes from 21 cancer types, with breast as the largest dataset [84]. This database is a repository for the meta-analysis based on the discovery and validation of biomarkers from the cancer survivors [85].

### Prognostic and meta-analysis of AURKA in BC (PrognoScan)

PrognoScan is a web-based tool for meta-analysis of prognostic value of genes. With its relatively simple user interface, it correlates gene expression with patient prognosis on the available cancer microarray datasets. Advantages of this tool include its large collection of publicly available cancer microarray datasets with clinical annotation and the assessment of the biological relationship between gene expression and prognosis. Furthermore, it utilizes the minimum *p*-value approach for classifying patients for survival analysis that evaluates the optimal cutpoint in continuous gene expression estimation without prior biological knowledge or assumption and therefore, enables systematic meta-analysis of numerous datasets [86]. This tool assessed at http://dna00.bio.kyutech.ac.jp/PrognoScan/ was used to evaluate AURKA as a potential biomarker and a possible BC therapeutic target.

### AURKA interaction analysis (STRING and Cytoscape)

The Search Tool for the Retrieval of Interacting Genes (STRING) database accessed at http://www.string-db.org/ provides extensive view of pre-existing, predicted interactions, and their associated proteins. To explore the co-expressed protein alongside AURKA gene, the proteins that associates with the AURKA gene was predicted and visualized by Cytoscape at http://www.cytoscape.org/. The output of this analysis is an interaction network showing various interacted genes as nodes. The AURKA node was taken as the “hub” genes in the protein network at a confidence level of 0.90. Additionally, the visual display was then adjusted (nodes, edges, and network) using appropriate layout and plugins to achieve a less dense/ clustered image. An additional interaction was further produced to evaluate their specificity to BC.

### Functional enrichment and pathway analysis

Standard gene IDs for a list of genes identified by STRING associated with AURKA were used as input in database for annotation, visualization and integrated discovery (DAVID database) in order to examine the gene functions and disease pathways by annotation of the protein partners alongside AURKA gene. This database was accessed at https://david.ncifcrf.gov/summary.jsp. The web-based tool displays the annotation summary results with several options. For the link gene-disease associations, the GAD_DISEASE was prioritized. The observed result ranked the AURKA-associated genes in different cancer types and processes with the special emphasis on the BC for this study. The gene ontology and pathway analysis were further analysed using this database.

### Dataset selection and docking study

#### Datasets

MiRTarBase v8.0 at http://mirtarbase.mbc.nctu.edu.tw/ is a freely available web-based tool for microRNA-gene interactions. These interactions were experimentally validated through molecular assays; MiRTarBase contains the largest microRNA to targets interactions (MTIs) datasets. Additionally, this tool provides the up to date MTIs collection which are cross referenced with other related databases [87]. The target microRNA sequence of AURKA was predicted by miRanda v10. This tool was used to retrieve the microRNA, and microRNA-target interaction sequences for downstream analysis.

The TargetScanHuman v7.2 is a microRNA target prediction tool that predicts the molecular targets for microRNAs that matches the 8mer, 7mer, and 6mer conserved regions in the seed region of each microRNA (http://www.targetscan.org/vert_72/) [88]. TargetScanHuman was used to confirm the target of the microRNA of interest.

RNAfold database at http://rna.tbi.univie.ac.at//cgi-bin/RNAWebSuite/RNAfold.cgi was used to predict the secondary structures, the minimum folding energy and doc-bracket notations of the microRNA and microRNA-AURKA duplex with the command line: RNAfold -p -d2 --noLP < sequence1.fa > sequence1.out. Furthermore, RNA-composer, a fully automated RNA structure modeling server at http://rnacomposer.cs.put.poznan.pl/ was used to generate a 3D PDB file format of the doc-bracket notations [89]. The crystal structure of (hAgo2) (PDB ID: 4F3T; resolution: 2.25 Å) was retrieved through the Protein Databank (PDB) for docking analysis.

### Protein preparation, docking, and visualization analyses

The molecular docking analysis of AURKA and microRNA of interest was performed by Schrodinger suite, PATCHDOCK, and Discovery Studio Visualizer (DSV) following the approach described by Fadaka *et al.* [90].

### Statistical analysis

The graph pad prism 6.0 software was used to analyze all the data in this study. Specifically, ANOVA and Student’s *t*-test were utilized to compute the clinical data between tests and controls. The ratio of the groups was correlated by the chi-square test. The odds ratio and 95% confidence interval were determined to assess the relationship between the gene of interest and BC risk through logistic analysis. All values were considered statistical significant at *p* < 0.05.

## CONCLUSIONS

The expression level of AURKA was significantly upregulated in patients with BC. Aberrant AURKA expression was found to be associated with poor prognosis of BC. The interactions observed within the distance of 3.5 Å and the hydrogen bonds within the distance of 2.0 Å were supportive that AURKA regulation through the AGO protein could be driven by miR-32-3p. These observed interactions are crucial to protein folding, stability and in the binding of targets. Based on the past and recent research on the roles of ncRNAs in the field of cancer, it can be inferred that therapeutics associated with microRNAs could provide desired therapeutic outcomes in cancer patients and related diseases. This study therefore, provides insight into the mode of AURKA regulation by miR-32-3p in BC. Formulations with ncRNAs can be more effective as therapeutic approaches and may represent a novel therapeutic intervention in BC and other cancer subtypes.

## Abbreviations

BC: Breast cancer
OS: Overall survival
PPS: Post-progression survival
DMSF: Distant metastasis free survival
RFS: Recurrence-free survival
hAGO2: Human Argonaute protein-2
AA: Amino acids
RISC: RNA induced silencing complex
H-bond: Hydrogen bond
BP: Biological process
MF: Molecular function
CC: Cellular component
